# Continuous post-disaster physical rehabilitation: a qualitative study on barriers and opportunities in Iran

**DOI:** 10.5249/jivr.v11i1.1036

**Published:** 2019-01

**Authors:** Ghasem Mousavi, Davoud Khorasani-Zavareh, Ali Ardalan, Hamidreza Khankeh, Abbas Ostadtaghizadeh, Mohammad Kamali, Gholamreza Raissi

**Affiliations:** ^*a*^Department of Health in Emergencies and Disasters, School of Public Health, Tehran University of Medical Sciences, Tehran, Iran.; ^*b*^Safety Promotion and Injury Prevention Research Center, Shahid Beheshti University of Medical Sciences, Tehran, Iran.; ^*c*^Department of Health in Emergencies and Disasters, School of Public Health and Safety, Shahid Beheshti University of Medical Sciences, Tehran, Iran.; ^*d*^Health in Emergency and Disaster Research Center, University of Social Welfare and Rehabilitation Sciences, Tehran, Iran.; ^*e*^Department of Clinical Science and Education, Karolinska Institutet, Stockholm, Sweden.; ^*f*^Iran University of Medical Sciences, School of Rehabilitation Sciences, Rehabilitation Basic Sciences Department, Tehran, Iran.; ^*g*^Neuromusculoskeletal Research Center, Physical Medicine and Rehabilitation Department, Iran University of Medical Sciences, Tehran, Iran.

**Keywords:** Rehabilitation, Earthquake, Disability, Disaster, Iran

## Abstract

**Background:**

Internationally, inclusion of physical rehabilitation services during early disaster response is relatively new. The aim of the study was to gain an understanding of disaster relief physical rehabilitation in Iran.

**Methods:**

A qualitative study design was employed and sixteen semi-structured interviews were conducted for data collection. Content analysis was used for data analysis. The participants in this study were purposively selected among people who experienced the Bam (2003) and Varzaghan (2012) earthquakes.

**Results:**

Three main themes were explored including: indispensable intervention, barriers to continuous in-tervention and opportunities for intervention. Almost all participants reiterated the importance of effective physical rehabilitation services during disasters. Some participants mentioned significant barriers for delivering such services in the context of Iran. The lack of an effective responsible body, weak disaster-related competencies and under-prioritization by government were among other barriers. On a more positive note, some interviewees talked about national programs that could facilitate service delivery.

**Conclusions:**

Providing disaster relief physical rehabilitation has faced many barriers in Iran. However, there are some facilitators in the country that could help provide these services. Finally, the feasibility of post-disaster physical rehabilitation services delivery completely depends on the current national rehabilitation system.

## Introduction

In total, 41 million Iranians inhabitants were affected by natural disasters between 1994 and 2013. This puts Iran among the top 10 countries with the highest absolute number of affected people over the past 25 years or so.^1-3^ During this period, Iran experienced several earthquakes, including the Bam earthquake in 2003 which is known as the most fatal natural disaster in Iran, and the Varzaghan earthquake in 2012. Death, disability and injuries are among the human impacts of disasters, which lead to 2-3 times more injuries than deaths.^4^ In term of injuries, fractures, blunt trauma, head trauma and spinal cord injuries are common in disasters.^3-6^ Proper medical response to disasters is critical in saving lives and treating injuries.^5^ The ultimate goal of medical intervention is the recovery of those injured so that they can attain an optimal level of independence.

Rehabilitation is considered a main healthcare strategy in some literature.^6^ It refers to a process that helps individuals with health conditions to reach and maintain optimal independence, and includes measures that restore the current function level for persons with probable disabling.^7^ Medical response in disasters is usually limited by various challenges such as managerial, human, technical, and financial problems.^8^ Rehabilitation in disasters, as a part of the whole medical response, encounters more challenges particularly in the early intervention of post-disaster impacts, as it is a new concept in disaster management.^9,10^ In 2005, the World Health Organization (WHO) emphasized the importance of rehabilitation during disasters. Accordingly, two groups of individuals benefited from disaster rehabilitation intervention; persons with existing disabilities, and people with severe injuries.^7^ WHO also point out that in addition to the affected populations, relief workers are also at risk of injury in the aftermath of natural disasters.^11^

Although most of the current literature focuses on the importance of physical rehabilitation during disasters, few studies explain how these services should be provided. According to WHO guideline, the functional recovery of the severely injured is complicated and requires the coordination of multiple professionals and treatment of secondary medical sequences.^12^ Moreover, a review of literature points out the challenges in providing effective disaster rehabilitation interventions.^13^ In addition, WHO released a guidance document in 2016^10^ which proposed to add rehabilitation specialists to the Emergency Medical Teams (EMTs) and outlined the minimum standards for rehabilitation in disasters.

There are very few studies in Iran and other low- and middle-income countries (LMICs) indicating how these services are provided. According to a study done after the Bam earthquake, the medical rehabilitation process was hampered by insufficient or ignored information.^14^ This study therefore aims to outline the barriers to and opportunities of the disaster physical rehabilitation services in Iran.

## Methods 

We used a qualitative approach through content analysis of semi-structured interviews with the providers and receivers of physical rehabilitation services after the Bam and Varzaghan earthquakes.

***Data collection and participant selection***

Purposive sampling was used for participant selection. The selection process was based on three criteria: physical rehabilitation service delivery in the affected area, the service receiver, and rehabilitation administration. All interviews were conducted between April 19 and June 30, 2015. To ensure comprehensive data collection, service deliverers as well as service receivers were interviewed. All interviews were performed in Persian except for those conducted with people who used physical rehabilitation services after the Varzaghan earthquake. Their interviews were in Turkish. One of the participants was a child and his father responded to the questions. Every interview was recorded and then transcribed verbatim. Initially, all interviews were transcribed in Farsi and then translated into English. The interviews were based on general questions about the participants’ rehabilitation related experiences and observations during the earthquakes. The principle investigator conducted 16 individual in-depth interviews. The interviews lasted between 30 to 60 minutes and were conducted at home (n=3), at the workplace (n=10) as well as at other places including hospitals and rehabilitation clinics (n=3). Interviews were recorded with consent and later transcribed immediately. Participant selection continued until data saturation was reached and the authors realized that new participants would not provide any new data.

Because the aim of the study was to investigate different points of the delivery of physical rehabilitation services during natural disasters, the researchers decided to conduct the interviews with participants who had different experiences. In the case of the Bam earthquake, only physical rehabilitation professionals were interviewed. Persons with disabilities and injuries were selected from people living in areas that were affected by the Varzaghan earthquake. Otherwise, physical rehabilitation providers were selected among those who experienced the Bam earthquake because the services provided in Bam were relatively broad and comprehensive.

***Data analysis***

The inductive approach was used for content analysis. ^15^ At the beginning, the texts were read several times and the meaning units that describe a specific experience were highlighted; then, codes were generated through the headings. Then, 48 sub-categories were obtained via codes and were grouped in order to reduce the number, and in the next step, 11 categories were generated and finally, three main themes were created.

***Ethical consideration***

The study gained ethics approval from the Ethical Committee of Tehran University of Medical Sciences and the approval letter number is: IR.TUMS.REC.1394.145.

***Trustworthiness***

Regarding the credibility of this study, the principal investigator (GHM) has many years of experience in the field of rehabilitation and disaster. He also used trust for data collection and analysis. Also, the research findings have been presented to four experts with experience of field and research work in this area, as a means of expert check, and they were asked to approve the data validation. In this way, participants’ approval has also been used, by means of member check. For dependability, the first step was to conduct interviews in a relaxed environment and all interviews were fully recorded. In fact, an attempt was made to record the voice of the interviewee fully and transparently carefully.

## Results

Table 1 shows the characteristics of the participants in the interviews.

**Table 1:** Characteristics of the study participants.

		Rehabilitation specialist	Physician	Disabled / Injured	Administrator
**Sex**	**Female**	0	0	3	1
	**Male**	4	2	1	5
**Age**	**Under 30**	0	1	1	0
	**30-45**	3	1	2	2
	**46-60**	1	0	0	4
	**Upper 60**	0	0	1	0

Table 2 shows themes, categories and sub-categories revealed through content analysis of the study interviews. Three subjects were considered as the main themes: 1) indispensable intervention, 2) barriers to sustainable intervention and 3) opportunities for intervention.

**Table 2:** The main themes, categories and sub-categories revealed through content analysis of the interviews.

Themes	Sub-themes	Codes
		Importance of assistive devices for users
		Permanent and temporary users
	The increasing unmet needs for rehabilitation	Absence of assistive devices
		Continuity of physical rehabilitation services
		Leave behind persons with disabilities
		Proper immobilization
**Indispensable intervention**	Physical rehabilitation in pre-hospital care	Consultation with rescue and relief workers
		Triage assistance
		Management of simple complications
		Proper consultation for patients
	Physical rehabilitation in hospital care	Immediate rehabilitation programs
		Injuries from vertebral column trauma
		Injuries requiring amputation
		Rehabilitation not in focus in national health system
		Lack of responsibility
		Shortage of specialists and resources
	The lack of effective responsible body	Poor link between medical specialists and rehabilitation
		Accessibility problems
**Barriers for continuous intervention**		Low general knowledge about rehabilitation
		Lack of planning
	Disaster-related competencies	Lack of practical education for students
		Resistance of rehabilitation specialists
		Services not free of charge
	Under-prioritization by government	Refusal to accept by disaster managers
		Not developing guidelines and teams
		Coordination among organizations
		Role of Iranian Red Crescent Society
	Recent developments in rehabilitation	Role of National Welfare Organization
		Role of Health Ministry
		Transporting severe disabled to facility
**Opportunities for intervention**		Family was unable to support
		International aid
	National and international NGOs, volunteers and private sector	NGOs
		Volunteers
		Private sector
	Community and Family Based Rehabilitation	FBR is conducted at national level
		CBR covers rural districts

***Indispensable intervention***

The three categories created in the main theme included: The increasing unmet needs for rehabilitation; physical rehabilitation in pre-hospital care; and physical rehabilitation in hospital care. All participants emphasized the importance and effectiveness of physical rehabilitation with regards to their experience and background.

***The increasing unmet needs for rehabilitation***

Some persons with disabilities benefited from assistive devices, such as wheelchairs, crutches and walkers, and some were completely dependent on such devices for mobility. Also, some people had orthotics and prosthetics or depended on other equipment to stay alive. Due to their dependence on a device, all of these people were more vulnerable in disasters and emergencies. One of the participants with a leg brace mentioned:

 “This device is essential for me because without it, my speed and reaction are reduced”.

In the event of a disaster, assistive devices might be lost or damaged. Not only did persons with disabilities use assistive devices, but there were also people using such devices for a short time as a result of temporary mobility limitations.

 “Some braces were repaired by local people, but some other braces needed experienced people who needed to come from Tabriz”.

Some of the participants emphasized the importance of other kinds of highly needed accessories. These included air mattresses and diapers, which participants said were critical in the life of persons with severe disabilities. Almost all participants accepted that the distribution of assistive devices was critical in the affected areas. Some participants believed that responsible organizations should keep reserves of such devices so that they can be distributed at the right time.

 “Rehabilitation and disability related accessories usually did not include relief packages, and this problem results in serious health problems for the permanent users of the accessories”.

Some participants talked about their experience of providing relief to people with disabilities during earthquakes and in the immediate aftermath. In such situation, people need specialist support from rehabilitation experts, because other people in the affected areas are not familiar with the specific conditions and needs. People with disabilities are more vulnerable due to cognitive and motor constraints than others and need to be transferred to specialized centers. One of the participants said:

 “I sent two disabled people to a residential center in Ahhar city. Because we had strong after-shocks one month after the earthquake and disabled people couldn’t stay at home. For this reason they couldn't run outside when the ground started shaking”.

Some participants who experienced the Varzaghan earthquake stated that regular referrals to rehabilitation therapy programs were discontinued after the earthquake. One participant was a hemiplegic child who was less than a year old at the time of the Varzaghan earthquake. His father responded to questions.

 “We didn’t know that he had a problem. No one came here”.

He was angry and upset as he felt that if specialists could have diagnosed his child’s problem in time and given him proper consultation, he could have followed his therapy program properly.

***Physical rehabilitation in pre hospital care***

Some of the participants witnessed a great number of those injured in the Varzaghan earthquake being brought to hospital by private vehicle. They believed that people with injuries came to the hospital without triage. They also mentioned that health professionals, such as rehabilitation specialists, could play a role in triage and properly immobilize the injured limbs of victims prior to transportation to hospital.

Rehabilitation specialists, based on their expertise, can train rescue and relief workers in the preparedness phase and can serve as consultants during early the response phase as well.

 “In my opinion, it is better for rehabilitation specialists to teach the rescue and first aid trainers the proper ways of transporting injured persons to hospital. Regarding their critical role in rescue and transportation of injured people after disasters, rehabilitation specialists should be trained in order to improve their life-saving skills”.

Rehabilitation specialists, because of their great knowledge and expertise, can participate actively in the early assessment and immobilization training given to educators, search and rescue workers and even the general public.

***Physical rehabilitation in hospital care***

Some of the participants believed that rehabilitation specialists, such as physiotherapists and occupational therapists, would be of “great help” in the management of the injured persons brought to the hospital with simple health problems. One of the participants said:

 “We were so busy and could not carefully examine the injured people, and made a mistake in fracture diagnosis. The presence of a physiotherapist can be of great help. He or she can manage patients with simple problems, such as ankle sprain or other conditions which need non-surgical intervention”.

Rehabilitation experts were able to counsel the victims in hospitals to continue and complete their treatment and remind them of how long rehabilitation takes. They can tell the injured people what actions should be avoided so that their injuries do not worsen. Many injuries do not require surgery, and rehabilitation specialists can handle these injuries. Also, rehabilitation experts have adequate knowledge and competence in the field of immobilization of fractures and in returning the range of motion in joints.

 “…For example, we had wrist trauma which needed fixation and immobilization for a week, and then we needed to work on the range of motion in the joint”.

According to participants’ experiences, a large proportion of patients were hospitalized with simple injuries, who took a lot of specialized doctors. Due to the low number of medical specialists and the importance of their presence in cases with severe head injuries requiring surgical intervention, simple hospital injuries could be managed by rehabilitation practitioners.

 “There were too many patients and only a few health personnel. We had to focus on one patient to keep them alive and avoid multi trauma…you had to splint the fracture for the patient but you had no time to talk to them and give them advice. If the rehabilitation team was present, they could have provided consultation for the patient to let them come back to continue the process of treatment. Most of our time was spent on serious patients”.

***Barriers to continuous intervention***

The main theme in disaster physical rehabilitation relief in Iran was generated by three categories: the lack of an effective responsible body; weak disaster-related competencies; and under-prioritization by government. Physical rehabilitation relief during disaster cannot be separated from the current rehabilitation system in the country.

***The lack of an effective responsible body***

Most of the participants believed that the cause of the many problems regarding rehabilitation in Iran was the lack of an effective responsible body. According to most participants, this problem has affected the country's rehabilitation system not only in emergency situations but also under normal conditions as it undermines the system’s position and function with regard to other aspects of health. The main weakness is the lack of a defined organization with enough authority at the national level. Almost all participants expressed the aforementioned weakness. Moreover, according to the participants, in spite of several governmental organizations functioning in the field of rehabilitation, rehabilitation leadership is complicated and unclear.

 “Rehabilitation has no responsibility at national level……We have infrastructure-related problems in rehabilitation. We have no rehabilitation deputy in the Ministry of Health or even a general manager. When there is no section for rehabilitation services in the Ministry of Health, there will be lots of divisions and subdivisions”.

The main consequence of this situation is the absence of rehabilitation services in the national health system plan. This absence has consequences in society; rehabilitation service delivery becomes problematic during disasters as well as in more normal situations. Another problem, expressed by the participants, was the low coverage of rehabilitation at national level. Rural areas were greatly affected by the Varzaghan earthquake, and had no rehabilitation facilities before the earthquake. Some participants who were injured during the earthquake could not use the physical rehabilitation services optimally.

 “I didn’t know what electrotherapy (physiotherapy) was. My doctor didn’t inform me at first about receiving electrotherapy. I went after three months of the earthquake incident…my family couldn’t take me to physiotherapy because of the long distance between our village and Tabriz, and I was required to go there several times".

Another participant who was injured as a result of earthquake said:

 “I could have easily gone for physiotherapy if physiotherapy was near our village and certainly my foot would have been better”.

A number of participants talked about the remoteness of the rehabilitation centers. This problem has caused some injured people not to complete their rehabilitation sessions. This problem has caused many injured people not to have access to rehabilitation services.

 “One of the problems with rehabilitation is the distance between patients and the hospital. There should be a center close to the patients”.

***Weak disaster-related competencies***

Every specialist needs some skills to work in the disaster field. Rehabilitation specialists are never trained for disasters and emergencies at university. Moreover, most of the specialists believe that rehabilitation isn’t an important issue.

 “Practical curriculums have been removed from students’ education”.

Many of the specialists are reluctant to work in affected areas. Based on responses from the participants, this reluctance is due firstly to a lack of training for disaster affected areas, and secondly a lack of skills for rehabilitating injured people.

 “But, unfortunately, as no good education and training are provided to our colleagues in universities about the definition of rehabilitation, they don’t come”.

Their unwillingness to attend the disaster areas, especially in the early days, is linked to the lack of preparedness. These experts do not therefore have a proper understanding of the real needs in these areas which prevent them from doing their job properly.

***Under-prioritization by health ministry***

Traditionally, rehabilitation follows on from medical treatment, so it is not included at prevention level. As expressed by some participants, rehabilitation isn’t needed in the early days of the disasters. The Ministry of Health and Medical Education has no good plan in emergency-related activities regarding rehabilitation.

 “Priorities of Health Ministry are more surgically oriented in natural disasters”.

Because of the low priority, lower resources are allocated to rehabilitation. Currently only medical treatment is free of charge in hospital, and disable people are required to pay for physiotherapy. Some participants stated that government organizations are moving toward healthcare rather than rehabilitation. They believed that rehabilitation was costly and therefore not a priority for governmental organizations.

 “Red Crescent Society should organize rehabilitation teams for disasters; the society gives low priority to rehabilitation services”.

***Opportunities for intervention***

Participants believed that, in spite of the barriers, there are considerable potential in the national rehabilitation system, including recent developments in rehabilitation, national and international NGOs, volunteers and the private sector and Community and Family Based Rehabilitation.

***Recent developments in rehabilitation***

Based on the responses, the rehabilitation sector is well developed in the country, relatively speaking. Every year, a large number are graduated in rehabilitation studies. Rehabilitation in Iran has a relatively long history and there are rehabilitation centers in all provinces that provide specialized services to those in need.

“Almost 1000 facilities function at country level. These government-supervised facilities provide both day and residential care for people with disabilities”.

According to some participants, these facilities can provide considerable help during emergency evacuation. However, one of the participants doubted these facilities have been prepared well. The participants believed that residential centers have sufficient resources and equipment. They also said that the centers have experts to manage persons with severe disabilities.

 “… In my opinion, persons with severe disabilities should be transported to residential settings, because families will not be able to care for them in the first days after the earthquake, they may have lost their families …”

In the experience of some participants, care of people with severe disabilities in hot weather and under a tent was “very difficult” and “not healthy”. As a result, they believed that the residential facilities were the best places for them.

***National and international NGOs, volunteers and the private sector***

In addition to governmental organizations, nongovernmental, private and volunteer institutions also provide skilled rehabilitation services to people with injuries and disabilities.

 “A private sector institution agreed to provide some braces for people with injuries and disabilities after the Bam earthquake” said one participant who provided artificial limbs in Bam.

Some participants expressed that, in their experience, benefactors and charities were even ahead of government agencies when it comes to meeting the needs of people with injuries or disabilities. They stated that they had been surprised by benefactors who, for example, had bought mattresses for wounded people and had donated money to a hospital. The participants considered volunteers to be highly capable. If organized properly, such people can help with the development of rehabilitation programs in disaster affected areas alongside nongovernmental organizations.

According to some participants, in addition to Iranian charities, a number of international non-governmental organizations were also present in the disaster areas (for example, the city of Bam) and were engaged in rehabilitation activities.

 “There were donations from NGOs from foreign countries such as France”.

Some participants referred to the advocacy role of volunteer organizations that could influence decision-makers to ensure policies and programs for providing the services.

***Community and Family Based Rehabilitation***

Participants informed us that there are currently various rehabilitation programs in the country. Community- and family-based rehabilitation is one form this kind of program takes.

In Iran, the national governmental welfare and rehabilitation organization (Behzisti) is legally responsible for community- and family-based rehabilitation. Community-based rehabilitation (CBR) focuses more on rural regions and family-based rehabilitation (FBR), is relatively new, and relies on family members and close relatives to support people with disabilities. A participant mentioned: according to another participant, FBR has no extension, as CBR has, but it does have the capacity to deploy in order to meet the needs of persons with disabilities during an emergency. CBR teams could contribute to rehabilitation in villages. They can have a good role in identifying the people in need and providing the necessary services.

 “Facilitators of CBR first came during the earthquake and, because they knew the locations of people with disabilities, especially in villages, they were sent to villages to assess the needs …”, “the facilitators are voluntary local young women”.

## Discussion

This study is the first one of its kind in the Eastern Mediterranean Region and highlighted barriers to and facilitators of continuous disaster physical rehabilitation in Iran. The findings are divided in three categories; indispensable intervention, barriers to continuous intervention and opportunities of intervention. Since all the interviews were conducted in Iran, the findings are based on the strengths and weaknesses of the geographical context. However, the rehabilitation situation is quite similar in developing countries.

The findings explore the necessity for medical rehabilitation relief after a natural disaster such as an earthquake. According to our findings, the benefits of physical rehabilitation teams following a disaster were numerous, from medical help by rehabilitation professionals to meeting the disaster-related needs of persons with disabilities for rehabilitation services. The benefits of the participation of rehabilitation professionals in pre-hospital care are clearly presented in the article. This finding was confirmed by Gosney et al. as they proposed that rehabilitation professionals could function actively in the triage of injured people and play a significant role in pre-operative and post-operative care. ^16^ Some studies in recent years have directly assessed the effect of physical rehabilitation services on post-disaster impacts. Zhang et al. reported that rehabilitation intervention improved the long-term functioning of Sichuan earthquake survivors with severe injuries.^17^ Ni et al. showed that rehabilitation intervention alleviated mental disorders, as well as physical dysfunction in fracture victims of the Sichuan earthquake.^18^ Yongqiang et al. demonstrated that formal physical rehabilitation improved the functional outcomes in a series of spinal-cord-injury victims from the 2005 Sichuan earthquake.^19^ These all imply that rehabilitation intervention can be useful during medical and surgical treatment. Knowlton and others stated that amputation rehabilitation professionals helped during the preoperative phase in the choice of limb amputation level. ^20^ Also in line with our findings, Rathore et al. stated that although persons with disabilities, caused by natural disasters, required early and proper transportation from disaster area, injured people were often evacuated and transported to local health facilities by untrained people and first responders without rehabilitation knowledge. The authors believed that rehabilitation should not only be involved in intermediate and long-term care settings but should also be included in immediate disaster response.^21^ Moreover, all studies support the necessity and importance of physical rehabilitation relief during early disaster response and long-term intervention.

Another important finding concerns the barriers to rehabilitation services delivery during natural disasters. One of the most important findings of the study is that the delivery of physical rehabilitation services during disasters completely depends on the existing rehabilitation system in a community. In areas with no developed rehabilitation systems, the service delivery will be inadequate in time of disasters and emergencies. This finding was clearly stated by the World Health Organization (WHO) in 2005.^22^ In addition, a system approach also is needed to be considered for improving rehabilitation of the disaster victims, as another study in a similar disaster context has also indicated regarding a system approach to injury prevention and its severity. ^23,24^ This paper has focused on the direct relation between the effects of disasters on persons with disabilities and the situation regarding health resources and rehabilitation facilities. One of the problems is poorly developed infrastructure in affected areas. According to our findings, successful rehabilitation intervention during natural disasters completely depends on the rehabilitation resources of the community. WHO`s official paper acknowledged this finding, stating that “In a setting where resources are limited, the impact of disasters on these groups of people [people with disabilities] can be long-term and far-reaching”. ^22^ This was obviously realized during earthquakes in Pakistan (2005) and Haiti (2010).^10,25,26^ Early health response teams usually include medical and surgical professionals. Disaster health decision-makers do not think about rehabilitation services. From the present findings, this was probably because of the low rehabilitation knowledge of the decision makers and the popular belief that rehabilitation was not an emergent need. Reinhardt et al. reported that the lack of rehabilitation services delivery during early disaster response was due to “surgical bias” and a low level of rehabilitation knowledge among response team members.^13^ Disasters are serious issues and as such require rapid response. Effective disaster response, including by the health sector, is reliant on the knowledge and competence of healthcare providers.^11^ Based on the findings, rehabilitation sector needs to and plan separately for the delivery of services in disaster-affected areas. This finding is exactly in line with an WHO document, which states that rehabilitation for work in affected areas requires a separate plan to that of the emergency medical teams.^10^ This implies that the delivery of physical rehabilitation services during disaster depends on the existing rehabilitation system in a community which should be strengthened.

Besides the barriers, according to the findings, the country has some characteristics which can potentially facilitate the physical rehabilitation relief program. Relatively good human resources exist in the country. Also, high number rehabilitation facilities deliver professional services across the country. Both governmental and nongovernmental organizations provide services in the country. Comprehensive approaches accepted internationally, are implemented in the national rehabilitation system.

As mentioned above, the World Health Organization has confirmed that those countries with good resources have a good rehabilitation system.^22^ The findings of this study also indicate that the availability of rehabilitation resources and centers in Iran can facilitate the effective provision of rehabilitation services in disaster-affected areas. Based on the responses of professional participants, there is a very close correlation between the status of a country's rehabilitation system and its ability to provide rehabilitation services in the event of natural disasters. Therefore, it can be said that these facilities and resources in any country can be considered as an opportunity for providing rehabilitation services in areas affected by natural disasters. The study states that international non-governmental organizations may be present in the affected areas of Iran and can provide specialized rehabilitation services in the days immediately following a disaster. After the April 2015 Nepal earthquake, international organizations were present from the very beginning in coordination with the local government and provided a range of rehabilitation services.^27^ According to the findings of this study, the Community-Based Rehabilitation approach, which is currently being implemented at country level, especially in rural areas, can be of assistance in addressing disability issues in emergencies. Given the fact that the plan has a formal mandate in the country, the previous coordination agencies should make it possible to use the plan in the affected areas. The WHO has also addressed this issue in a book, in which the organization emphasized the role of the community-based rehabilitation approach in disaster management. It suggests that this approach helps people with disabilities to have better preparedness to deal with disasters, and improves disaster management plans.^28^

***Strengths and limitations of the study***

Despite the fact that this article was the first study in the field of rehabilitation in disasters in Iran, it was able to identify some of the hidden aspects of this area using the experiences of specialists, injured people and people with disabilities, but it had some limitations. One of the limitations of the study was a relatively long time between the experience of some of the participants and the interview time. Because of this, we used non-expert participants who used the services recruited from the people living in the Varzaghan area. Another limitation of the study was that since the study was carried out in the context of Iran, its results cannot be generalized to other countries. However, since qualitative study doesn’t aim to generalize the findings,^29^ the author also doesn’t claim that the findings can be generalized to other settings.

## Conclusion

Despite the necessity and importance of physical rehabilitation intervention during disasters, delivery of such services encounters great problems arising from a weak organizational structure and rehabilitation system in Iran. However, some national and international programs in operation can facilitate the utilization of physical rehabilitation during disasters and emergencies. Physical rehabilitation disaster relief can be included in the national disaster health response if the barriers can be broken down and the opportunities utilized.

**Acknowledgements**

We thank Iranian Handicap Society and all of the participants for the generous support to the study. This study has done as a part a PhD in disaster management at School of Public Health at the Tehran University of Medical Sciences.
